# Prolonged critical avalanche burial for nearly 23 h with severe hypothermia and severe frostbite with good recovery: a case report

**DOI:** 10.1186/s13049-024-01184-3

**Published:** 2024-02-12

**Authors:** Elisabeth Gruber, Rosmarie Oberhammer, Hermann Brugger, Elisa Bresadola, Matteo Avogadri, Julia Kompatscher, Marc Kaufmann

**Affiliations:** 1 Department of Emergency Medicine, Anaesthesia and Intensive Care, Hospital of Bolzano (SABES-ASDAA), Teaching Hospital of Paracelsus Medical University, Via Lorenz Boehler 5, 39100 Bolzano-Bozen, Italy; 2 HELI HEMS Service South Tyrol, Via Lorenz Boehler 3, 39100 Bolzano-Bozen, Italy; 3 Department of Anaesthesia and Intensive Care, Emergency Medicine and Pain Therapy, Hospital of Brunico (SABES-ASDAA), Teaching Hospital of Paracelsus Medical University, Via Ospedale 11, 39031 Brunico-Bruneck, Italy; 4 Institute of Mountain Emergency Medicine, Eurac Research, Via Ipazia 2, 39100 Bolzano-Bozen, Italy; 5 Aiut Alpin Dolomites Helicopter Emergency Medical Service, Pontives 24, 39040 Laion- Lajen, Italy

**Keywords:** Accidental hypothermia, Avalanche, Frostbite, Cold injury, Rewarming, Thrombolysis, Iloprost, Emergency medicine

## Abstract

**Background:**

Accidental hypothermia with severe frostbite is a rare combination of injuries with a high risk for long-term sequelae. There are widely accepted recommendations for the management of avalanche victims and for frostbite treatment, but no recommendation exists for the treatment of frostbite in severe hypothermic patients, specifically for the management of hypothermic avalanche victims presenting with frostbite.

**Case presentation:**

We present a case of a previously healthy, 53-year-old male skier who was critically buried by an avalanche at 2300 m of altitude at an ambient temperature of − 8 °C for nearly 23 h. The victim was found with the right hand out of the snow and an air connection to outside. He was somnolent with Glasgow Coma Scale 11 (Eye 4, Verbal 2, Motor 5) and spontaneously breathing, in a severely hypothermic state with an initial core temperature of 23.1 °C and signs of cold injuries in all four extremities. After rescue and active external forced air rewarming in the intensive care unit, the clinical signs of first-degree frostbite on both feet and the left hand vanished, while third- to fourth-degree frostbite injuries became apparent on all fingers of the right hand. After reaching a core body temperature of approximately 36 °C, aggressive frostbite treatment was started with peripheral arterial catheter-directed thrombolysis with alteplase, intravenous iloprost, ibuprofen, dexamethasone and regional sympathicolysis with a right-sided continuous axillary block. After ten months, the patient had no tissue loss but needed neuropathic pain treatment with pregabalin.

**Conclusion:**

The combination of severe accidental hypothermia and severe frostbite is rare and challenging, as drug metabolism is unpredictable in a hypothermic patient and no recommendations for combined treatment exist. There is general agreement to give hypothermia treatment the priority and to begin frostbite treatment as early as possible after full rewarming of the patient. More evidence is needed to identify the optimal dosage and time point to initiate treatment of frostbite in severely hypothermic patients. This should be taken into consideration by future treatment recommendations.

## Background

The probability of survival of avalanche victims depends on the duration of burial, airway patency and degree of injury [1]; the high mortality of approximately 50% [2] is attributed to asphyxia, trauma, and hypothermia [3]. Accidental hypothermia of mild and moderate degree is a frequent finding in avalanche survivors [2], but the occurrence of severe frostbite in a severely hypothermic, critically buried avalanche victim has been reported only once in the literature, unfortunately with significant tissue loss [4].

There are widely accepted recommendations for the management of avalanche victims [5–7] and for frostbite treatment [8], but no recommendation exists for the treatment of frostbite in hypothermic patients, specifically for the management of hypothermic avalanche victims presenting with third- to fourth-degree frostbite injuries [8]. Guidelines acknowledge that no studies examine concurrent hypothermia and frostbite, although there is general agreement that moderate and severe hypothermia should be treated effectively before treating frostbite [8].

## Case presentation

We describe a case of prolonged critical avalanche burial of nearly 23 h with severe hypothermia (initial core temperature 23.1 °C), first-degree frostbite of the left hand and both feet and third- to fourth-degree frostbite [8] of the right hand.

In January 2023, a 53-year-old male (height 180 cm, weight 76 kg, body mass index 23.4 kg/m^2^, ASA classification I [9]) set off alone on a ski tour in the Italian Alps during an avalanche risk level of three. He was buried by an avalanche at around 12 p.m. during ascent at approximately 2300 m above sea level. His family called the emergency services at approximately 6 p.m. Immediately, the alerted mountain rescue service began to collect information to determine the possible routes the victim might have been on and the areas that need to be searched. Several search and rescue (SAR) flights were performed during the night, including the use of an international mobile subscriber identify (IMSI) catcher. All night flights failed to locate the victim because of missing information about the site of the accident.

At the break of the next day, multiple avalanche sites were identified and thoroughly searched using a helicopter-mounted avalanche transceiver. After approximately 23 h of the presumed burial, a signal could be detected, and surprisingly, a moving hand was identified on the surface of the avalanche.

**Fig. 1:**
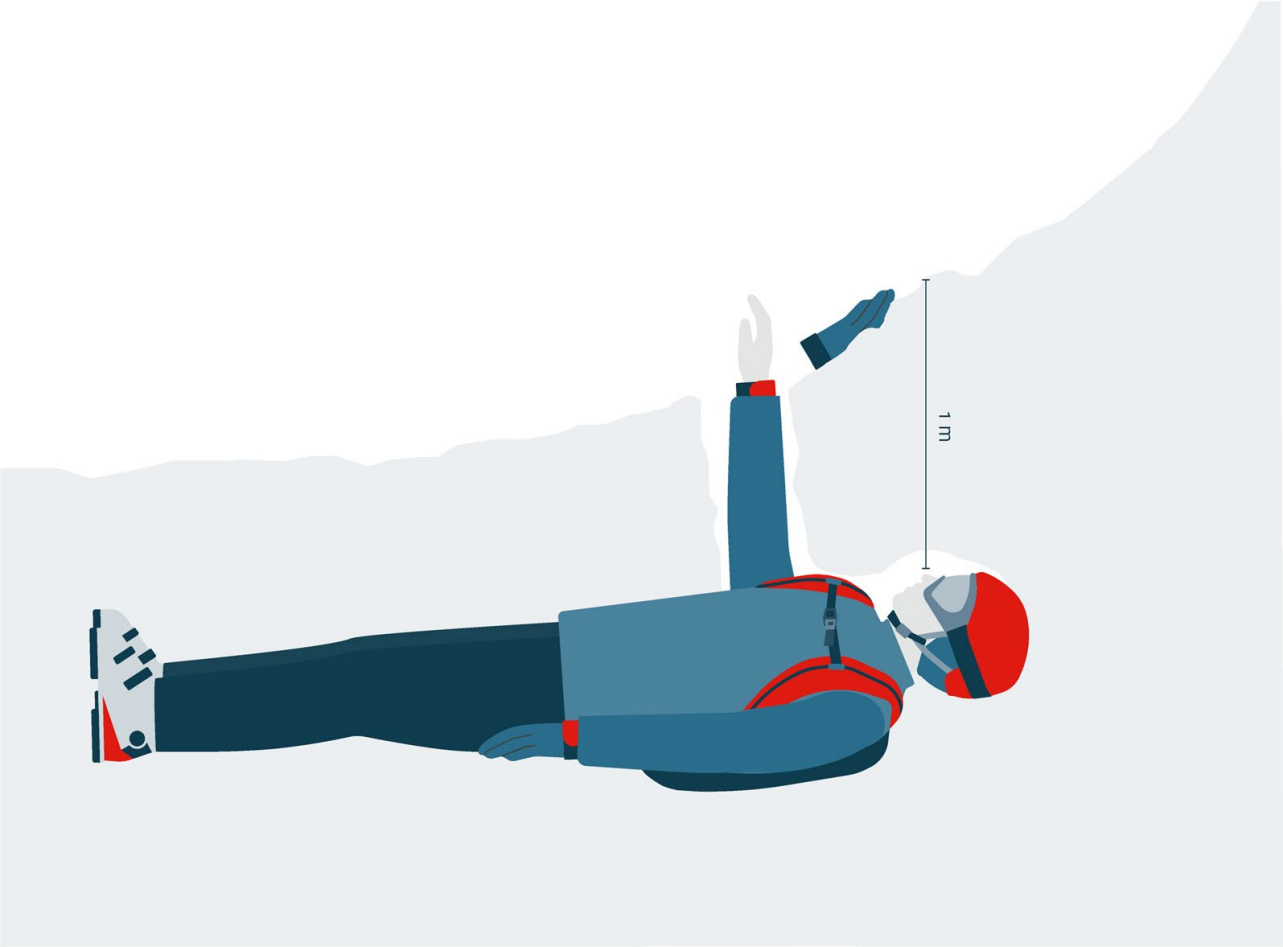
Patient’s supine position during avalanche burial. The ungloved right hand reached the outside and formed an air channel to mouth and nose which enabled the victim to breathe

On arrival of the rescue team at the burial site the victim was critically buried in a supine position with the head at a depth of approximately one meter. The right ungloved hand reached the outside through an air canal and was free of snow (Fig. 1). The rescuers identified a large air pocket in front of the mouth and nose that was connected to the outside and a patent airway. The victim was adequately dressed for the ambient conditions with cap, windbreaker, and gloves, except on the right hand. The ambient temperature was − 8 °C.

The body was extricated within a few minutes at 10.45 a.m. The patient had no major bleeding, was reactive to verbal stimuli, was agitated with myosis and a Glasgow Coma Scale (GCS) score of 11 (Eye 4, Verbal 2, Motor 5) and was spontaneously breathing (respiratory rate 6/min). Haemodynamic parameters were unstable with bradycardia 40–50 beats/min and a weak central but no peripheral pulse. The first core body temperature measured epitympanically with a thermistor-based probe (TTS-400 ICU Medical Temp Sensor Tympanic by Smiths Medical) was 23.1 °C.

The patient was gently extricated, immobilised on a vacuum mattress and wrapped in an active self-warming blanket (Mölnlycke^®^ BARRIER^®^ EasyWarm^®^). For the 25 min helicopter transport to the nearest level I trauma centre of Bolzano with extracorporeal membrane oxygenation (ECMO) capabilities, a mechanical chest compression device (Schiller Easy Pulse^®^) was attached but never activated.

**Fig. 2:**
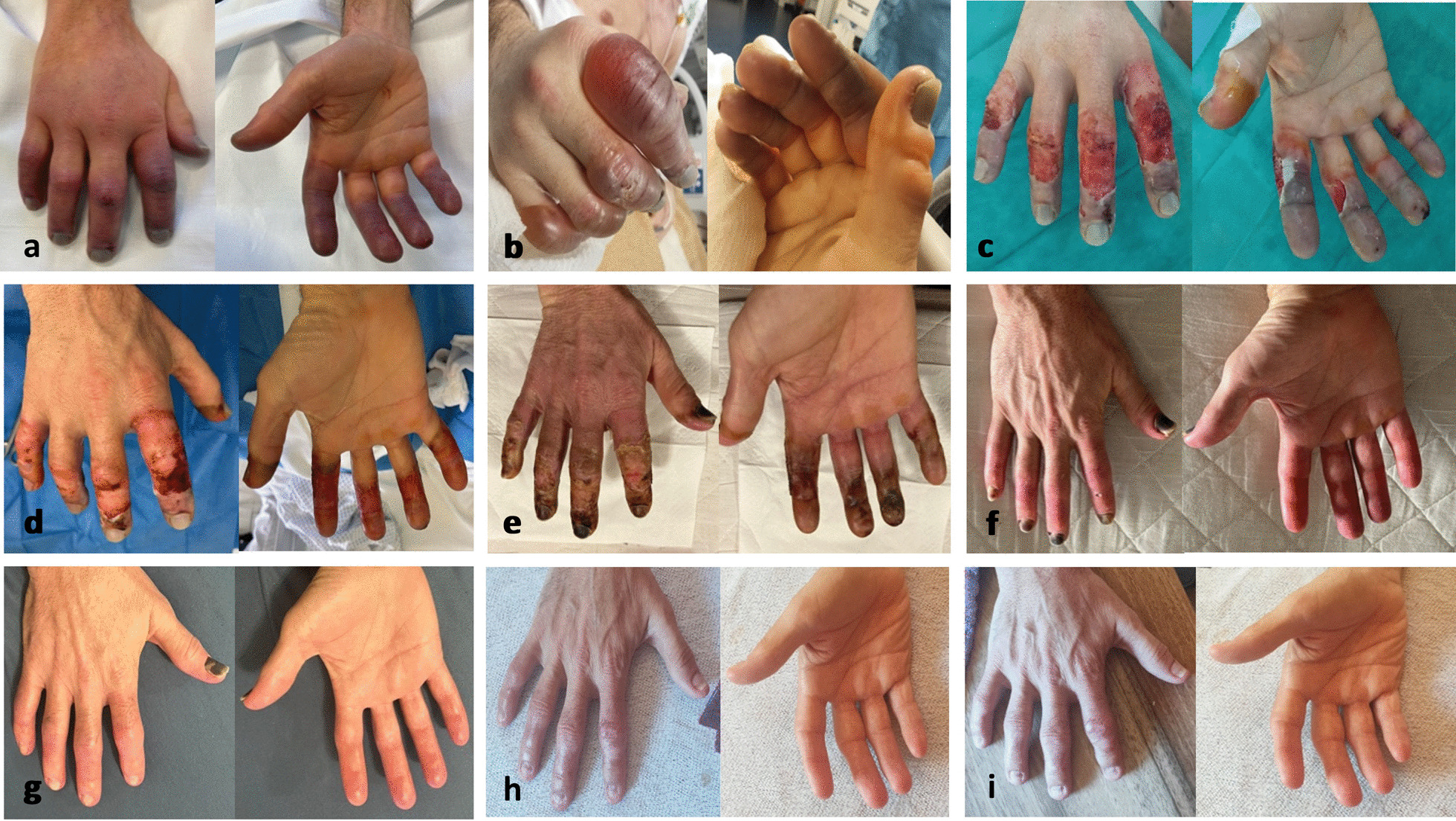
Progression of frostbite of the right hand. **a** = day 1 after rewarming to 36° C core temperature, **b** = day 4, **c** = day 7, **d** = day 10, **e** = day 27, **f **= day 49, **g** = 3 months, **h** = 5 months, **i** = 10 months

On admission at midday the patient was still somnolent with GCS 11 (Eye 4, Verbal 2, Motor 5), core body temperature measured in the bladder 26.4 °C, sinus tachycardia (114/min), normal blood pressure and tachypnoea (20/min). Noninvasive SpO_2_ was not measurable because of cold extremities. With external forced warm air rewarming (Bair Hugger™), normothermia (36 °C) was reached in four hours. During rewarming, the patient was not moved to avoid life-threatening cardiac arrhythmias. Before rewarming, all hands and feet were pale and cold to the touch. These signs of frostbite vanished on the left hand and both lower legs after rewarming. The right hand showed third- to fourth-degree frostbite on the entire second finger, the distal and middle phalanx and distal part of the proximal phalanx of the fifth finger and the areas distal to the proximal interphalangeal joints of the third and fourth fingers. Additionally, there was third-degree frostbite on the distal phalanx and second-degree frostbite on the proximal phalanx of the thumb (Fig. 2). In the total body computed tomography (CT) scan after rewarming, there were no signs of traumatic injury. For the treatment of rhabdomyolysis (CK > 22,000 U/l), continuous renal replacement therapy (CRRT) with CytoSorb^®^ (Aferetica) was performed for 24 h.

Frostbite treatment was started approximately 5 h after admission according to the local protocol with ibuprofen 6 mg/kg every 12 h, dexamethasone 0.1 mg/kg daily and iloprost (0.5 ng/kg/min increasing to 1.0 ng/kg/min) for five days. We established a continuous right-sided axillary block (Contiplex^®^ S Ultra 360 18G) for sympathicolysis with ropivacaine 0.2% 4 ml/h for 4 days. The patient received enoxaparine 6000 IE/day for thromboprophylaxis. After exclusion of contraindications and any compartment syndrome, catheter-directed thrombolysis (CDT) with alteplase via a retrograde cannula (Arrow R 22G) inserted in the right brachial artery was started approximately 4 h after rewarming with a bolus of 3 mg and continuous infusion of 1 mg/h for 6 h. A broad-spectrum antibiotic (amoxicillin/clavulanic acid 2.2 g × 3/d for 5 days) and antitetanic vaccine were administered.

On day 2, the frostbite lesions on the right hand seemed to improve, but 48 h after thrombolysis, haemorrhagic blisters appeared. Debridement of high tense blisters with risk of uncontrolled rupture was performed.

Also day 2, the patient suffered from diffuse musculoskeletal pain, and signs of compartment syndrome on the left shoulder appeared, probably caused by compression of the backpack straps during burial. No surgical treatment was needed.

On day 7, a triple phase bone scan with single photon emission computed tomography (SPECT/CT) imaging was performed to assess the ischaemic but viable bone regions and to allow an estimation of tissue loss. Immediately after administration of the 16 mCi of 99 m-Techentium methylene diphosphonate (99mTc MDP), perfusion and blood pool images revealed reduced uptake of the tracer in the distal parts of the second to fifth fingers of the right hand. The delayed phase images obtained 3 h after injection of the radiopharmaceutical revealed relatively increased tracer uptake of soft tissue from second to fifth finger and in the distal parts of the thumb.

On day 10, the patient was discharged with multimodal analgesia with nonsteroidal anti-inflammatory drugs, opioids, pregabalin and palmitoiletanolamide due to persistent nociceptive and neuropathic pain (mixed pain) in the right hand, left shoulder and right lower leg. Movement of the right hand was limited because of stiffness and pain. For posttraumatic stress disorder, the patient received preventive psychologic support and a tranquilizer.

After ten months, the patient reported a full functional recovery of all fingers of the right hand except for nail dystrophia (Fig. 2) but complained of hypoaesthesia and hypersensitivity to ambient temperatures below 14 °C. He continues treatment of neuropathic pain with pregabalin and resumed sports activity (cycling and hiking).

## Discussion and conclusions

We present a case of exceptionally long critical avalanche burial with severe accidental hypothermia and severe frostbite.

The combination of severe accidental hypothermia and frostbite is rare, and studies on both injuries as well as recommendations for concurrent treatment are lacking. It is widely accepted that moderate and severe hypothermia should be treated effectively before frostbite injury [8], and frozen tissue should be thawed as soon as circulation and core temperature have stabilized [8, 10, 11]. However, the pathophysiological interactions between hypothermia and frostbite are largely unknown, and it is unclear to what extent hypothermia treatment must be completed before frostbite treatment can be commenced. It is also not evident which drugs at what dosage can be administered for frostbite treatment [10]. Cold exposure leads to peripheral vasoconstriction [12], in hypothermia due to lowering core temperature < 35 °C to protect the core body organs from further heat loss and in frostbite due to direct cold exposure of mainly unprotected skin. In frostbite, heat loss below the tissue freezing point (− 0.5 °C) causes ice crystal formation in superficial and deep tissue [8]. Environmental temperature, wind chill factor, length of exposure, clothing, concomitant factors such as alcohol and drug abuse and psychiatric disorders influence the severity of frostbite lesions [13]. During skin cooling, the “hunting reaction”, an adaptive response with cycles of vasoconstriction followed by vasodilation, can be observed [14]. Cold exposure can damage neurons in the skin with loss of cutaneous sensations and decreasing or vanishing pain, with the risk of prolonging further cold exposure. Blood viscosity can increase, and blood flow to the affected tissue can be shunted away to maintain core temperature with possible future tissue cooling when cold exposure continues [10]. During tissue freezing, due to extra and intracellular ice crystal formation, the osmotic gradient changes, which causes electrolyte shifts, pH alterations, intracellular dehydration, and hyperosmolality, harming the cellular integrity of the affected tissue [15–17].

Human data about the efficacy of drugs in hypothermia are limited and based mainly on prehospital studies [5]. Hypothermia may alter the pharmacokinetic and/or pharmacodynamic parameters of drugs, resulting in therapy failure or toxicity [18]. Altered clearance seems to be the most important pharmacokinetic effect for drugs with a narrow therapeutic index [18]. Changes in the volume of distribution (VD) occur during cooling as well as during rewarming [18]. The vasoconstriction and blood redistribution from the extremities, intestine and solid intraabdominal organs to the heart and brain decreases VD with the risk of toxicity with the usually recommended drug dosages. On the other hand, vasodilation during rewarming increases VD with the risk of therapy failure [18]. Due to scarce data, experts suggest for the cardiac arrest situation to withhold adrenaline and other CPR drugs until the patient is rewarmed to a core temperature ≥ 30 °C, to double intervals between drug doses until 35 °C and thereafter to use a standard drug protocol [5]. In the case hypothermia is associated with frostbite, treatment with epinephrine may be harmful because of further vasoconstriction in the extremities [19]. Additionally, other vasoconstrictive agents, such as norepinephrine, can impair perfusion in the extremities. During rewarming, vasodilation in hypothermic patients occurs, and cardiocirculatory instability with the need for fluid replacement therapy to prevent shock is frequent [11].

On the other hand, in patients with frostbite, vasodilation and perfusion are optimised with iloprost [20] and sympathicolysis due to regional anaesthesia, and thrombolytics and other drugs better reach the frostbitten area after thawing.

Thrombolytics can be administered intravenously or intraarterially with similar limb salvage rates of 77.3% and 76.4%, respectively [21]. However, the intraarterial route can be challenging and makes sense only for severe frostbite treatment in 1–2 extremities, whereas intravenous administration can be easier to perform and is the first choice for frostbite on 3–4 extremities [22]. Each hour of delay of thrombolysis after thawing the severe frostbitten area results in a 28.1% decrease in limb/digit salvage [23].

Thrombolysis has a risk of bleeding [24], resulting in complications such as compartment syndrome, and requires changes in frostbite management in 8.4% of cases [25]. Especially in patients with concomitant traumatic injuries, hypothermia-induced coagulopathy with elevated bleeding risk should also be considered. On the other hand, the procoagulant effect with increased clot extension and formation during freezing requires treatment with fibrinolytics and unfractionated heparin (UF) or low-molecular weight heparins (LMWH) and iloprost [20]. In our case, we decided for LMWH for several advantages: easier route for administration (subcutaneous vs intravenous), more predictable anticoagulant effect without need of close laboratory tests, and less likelihood for immune-mediated thrombopenia with thrombus formation [26]. High-quality evidence for severe frostbite treatment is lacking. It is possible that the combination of iloprost and anticoagulation with LMWH augmented the digit salvage rate [24]. Cauchy et al. described a lower risk of digit loss in the iloprost group than in the iloprost and alteplase groups [27]. However, in the combination group, there were more severely frostbitten digits [27]. A randomised controlled noninferiority study to compare alteplase to iloprost efficacy is ongoing [28]. Unlike the strong correlation between treatment delay and limb/digit loss using thrombolytics in severe frostbite with no benefit after 24 h, iloprost benefit was evident even in delayed treatment up to 5 days [27, 29]. Due to these results and the low complication rate [24], iloprost seems to be an important pillar in frostbite treatment in hypothermic patients, especially when the time to reach normothermia and normalisation of coagulation is prolonged. For wound healing improvement [30] and its anti-inflammatory effects, dexamethasone was administered.

In our case, the severe frostbite on the right hand of the avalanche victim, just rewarmed to normal core temperature, was treated by all available means, i.e., intraarterial thrombolysis, LMWH, platelet anti-aggregation and vasodilation with intravenous administration of synthetic prostaglandin I_2_ analogon iloprost, vasodilation through sympaticolysis using continuous axillary block, anti-inflammatory and analgesic acting ibuprofen and opioids. We assume that this aggressive therapeutic approach may have saved the hand from extensive tissue loss. In an almost identical case, a young healthy avalanche victim lost all fingers of the left hand after 9 h of critical burial with the hand exposed to outside at low temperatures [4].

**Fig. 3:**
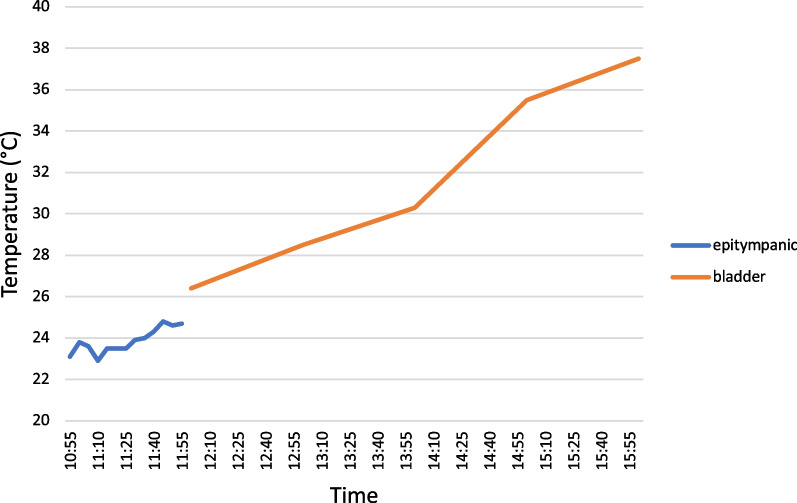
Core body temperature (Tc) in relation to time out of hospital and in hospital. Out-of-hospital rewarming with an active self-warming blanket (Mölnlycke^®^ BARRIER^®^ EasyWarm^®^), in-hospital rewarming with Bair H Hugger™. Epitympanic Temperature may be underestimated because of low ambient temperature (− 8 °C) [33, 34]

The first measured core temperature on site was 23.1 °C using an epitympanic probe. The low initial epitympanic core temperature measured during the out-of-hospital phase seems to be in contrast with clinical signs of the patient, responsiveness, and the presence of vital functions [31]. However, Pasquier et al. showed in a retrospective study a high variability of the core temperature with respect to the clinical stage (not-modified Swiss staging) [32]. In approximately 40% of 305 hypothermic cases the measured core body temperature did not correspond to the expected clinical stage. Specifically, in patients presenting with Swiss stage 2 (somnolent, not shivering) the lowest recorded core temperature was 22 °C. It is also known that epitympanic measurements may give false low values at low ambient temperatures if the ear probe is not well insulated against the outside [33, 34] as it was the case in our patient. There was a difference of 1.7 °C between the epitympanically measured temperature and the bladder temperature (Fig. 3). The first in-hospital measurement ot the bladder temperature was 26.3 °C. It should be considered that in hypothermic patients even the bladder temperature can give too low values when compared with the core temperature in the pulmonary artery [35, 36].

Rhabdomyolysis is a well-known and common problem after long immobilisation [37]. However, in our case, there are other causes contributing to the important crush syndrome of the patient: the compression of the snow, the continuous muscle activity of the right leg and right arm during burial and the hypothermia itself [38, 39]. Early treatment is the key for a good clinical outcome with different therapeutic options, including CRRT [37].

In conclusion, the combination of critical avalanche burial and severe frostbite is very rare, and treatment is challenging. This case represents successful management of severe accidental hypothermia, hypothermia-associated rhabdomyolysis and severe frostbite in an avalanche victim with good neurological recovery and no tissue loss.

In a severely hypothermic patient with severe frostbite, it seems essential that frostbite treatment commences as early as possible during or after rewarming to close-to-normal core body temperature. This should be taken into consideration by future treatment recommendations.

## Data Availability

The data used to support the findings of this case study are included within the article. The detailed data regarding the case presented are available from the corresponding author on reasonable request.

## Abbreviations

ECMO: Extracorporeal membrane oxygenation
CDT: Catheter-directed thrombolysis
CRRT: Continuous renal replacement therapy
CT: Computed tomography
GCS: Glasgow coma scale
IMSI: International mobile subscriber identify
LMWH: Low-molecular weight heparins
SAR: Search and rescue
SPECT: Single photon emission computed tomography
UF: Unfractionated heparin
VD: Volume of distribution
